# Association of concerns about developing Alzheimer's disease with higher amyloid burden and lifestyle behaviors in cognitively unimpaired older adults

**DOI:** 10.1002/alz70857_097819

**Published:** 2025-12-24

**Authors:** Francesca R Farina, Marc P Bennett, Josh D Grill, Reisa A. Sperling, Brian Lawlor, James W Griffith

**Affiliations:** ^1^ University of Chicago, Chicago, IL, USA; ^2^ Global Brain Health Institute, Trinity College Dublin, Dublin, Ireland; ^3^ University College Dublin, Dublin, Dublin, Ireland; ^4^ The UC Irvine Institute for Memory Impairments and Neurological Disorders, Irvine, CA, USA; ^5^ Harvard Medical School, Boston, MA, USA

## Abstract

**Background:**

Concerns about developing Alzheimer's disease (AD) are common. AD concerns have been shown to indicate pathological changes like amyloid accumulation in individuals with subjective cognitive decline. However, the extent to which AD concerns relate to such changes in cognitively unimpaired populations is unclear. Our aim was to investigate if AD concerns are associated with amyloid burden in cognitively unimpaired older adults, and how these concerns relate to lifestyle behaviors.

**Method:**

We analyzed screening data from the Anti‐Amyloid Treatment in Asymptomatic Alzheimer (A4) study, which is a randomized clinical trial conducted across 67 sites. Data were collected between April 2014 and December 2017 and analyzed in October 2024. Participants were cognitively unimpaired older adults (65‐85 years; Clinical Dementia Rating: 0 and/or Mini‐Mental State Examination (MMSE) score: ≥25) who underwent positron emission tomography (PET) imaging to assess cortical amyloid beta levels. Measures were screening demographics, lifestyle variables, APOE genotyping, AD concerns (Concerns about Alzheimer's Disease Questionnaire; CADQ), anxiety (State‐Trait Anxiety Inventory; STAI), depression (Geriatric Depression Scale; GDS), cognition (MMSE, Cognitive Function Index; CFI), and florbetapir amyloid PET. AD concerns were assessed prior to disclosure of amyloid or APOE status.

**Result:**

Of 4460 individuals (mean [SD] age, 71.29 [4.67] years, 59.4% female), AD concerns were elevated in: females compared to males (mean [SD] CADQ = 21.3 [4.62] vs. 20.34 [4.64]; *p* < .001), individuals with a parental history of dementia (21.71 [4.36] vs. 19.46 [4.83]; *p* < .001), APOEε4 carriers (21.56 [4.54] vs. 20.56 [4.68]; *p* < .001), and those who did not meet recommended guidelines for walking (21.16 [4.66] vs 20.83 [4.64]; *p* = 0.032) or sleep (21.14 [4.81] vs. 20.83 [4.59]; *p* = 0.039). AD concerns were associated with higher amyloid burden (*p* = 0.007) after adjusting for demographics, anxiety, depression, cognition, parental history and APOEε4. This model outperformed the reference model without AD concerns. The association between AD concerns and amyloid burden was stronger in APOEε4 carriers.

**Conclusion:**

AD concerns were associated with elevated amyloid, a core diagnostic AD biomarker. Assessing AD concerns could inform future cohort and intervention recruitment strategies.

